# Supplementary material for: Insecticide resistance and species diversity in Anopheles gambiae s.l. in Côte d’Ivoire
Source: PLoS One. 2025 Sep 30;20(9):e0332497. doi: 10.1371/journal.pone.0332497 (PMC12483214; doi:10.1371/journal.pone.0332497)
Supplement: S1 Table — (DOCX) [file pone.0332497.s001.docx]

**S2 Table.** Variation in allelic frequencies of *Kdr* L1014F and *Ace-1^R^* G119S mutations within members of *An. gambiae* s.l*.* by study area

|  |  |  | *Anopheles gambiae* | | | |  | *Anopheles coluzzii* | | | |
| --- | --- | --- | --- | --- | --- | --- | --- | --- | --- | --- | --- |
|  |  |  | Genotypes | | | % |  | Genotypes | | | % |
| Types | Capture methods | Zones | RR | RS | SS | Frequency |  | RR | RS | SS | Frequency |
| *Kdr* L1014F | Larval catches | Savannah | 39 | 0 | 0 | 100.0 |  | - | - | - | - |
|  |  | Transition | 12 | 0 | 0 | 100.0 |  | 43 | 49 | 19 | 83.3 |
|  |  | Forest | 21 | 8 | 3 | 78.1 |  | 34 | 38 | 12 | 63.1 |
|  |  | Total | 72 | 8 | 3 | 91.6 |  | 77 | 87 | 31 | 61.8 |
|  | Adult caches | Savannah | 41 | 0 | 0 | 100.0 |  | 1 | 0 | 0 | - |
|  |  | Transition | 5 | 0 | 0 | 100.0 |  | 41 | 47 | 22 | 58.6 |
|  |  | Forest | 10 | 8 | 9 | 51.8 |  | 4 | 8 | 2 | 57.2 |
|  |  | Total | 56 | 8 | 9 | 82.2 |  | 45 | 55 | 24 | 58.5 |
| *Ace-1^R^* G119S | Larval catches | Savannah | 4 | 17 | 17 | 32.9 |  | - | - | - | - |
|  |  | Transition | 1 | 0 | 11 | 8.3 |  | 3 | 28 | 77 | 15.7 |
|  |  | Forest | 7 | 9 | 15 | 37.1 |  | 1 | 15 | 69 | 10.0 |
|  |  | Total | 12 | 26 | 43 | 30.9 |  | 4 | 43 | 146 | 13.2 |
|  | Adult caches | Savannah | 1 | 19 | 19 | 26.9 |  | 0 | 0 | 1 | - |
|  |  | Transition | 0 | 2 | 3 | 20.0 |  | 2 | 32 | 76 | 16.4 |
|  |  | Forest | 2 | 14 | 11 | 33.3 |  | 0 | 8 | 6 | 28.6 |
|  |  | Total | 3 | 35 | 33 | 28.9 |  | 2 | 40 | 83 | 17.6 |
