# Supplementary material for: Insecticide resistance and species diversity in Anopheles gambiae s.l. in Côte d’Ivoire
Source: PLoS One. 2025 Sep 30;20(9):e0332497. doi: 10.1371/journal.pone.0332497 (PMC12483214; doi:10.1371/journal.pone.0332497)
Supplement: Attachment — Submitted filename: Response to Reviewers_PONE-D-24-56516.docx [file pone.0332497.s002.docx]

Abidjan, August 13^th^ 2025

**Rosine Zlanneu WOLIE, PhD**

Université Nangui Abrogoua (UNA), Abidjan

Vcetor Control Product Evaluation Centre (VCPEC), Institut Pierre Richet (IPR), Bouaké

BP 1500, Bouaké

Côte d’Ivoire

Phone : (+225) 0748532551

E-mail: [wolierosine@yahoo.fr](mailto:wolierosine@yahoo.fr)

**To: The Editors of PLOS ONE**

**Subject: Resubmission of Revised Manuscript - PONE-D-24-56516**

Dear Editors,

I hope this message finds you well.

First of all, we would like to apologize for the delay in submitting our revised manuscript titled “Insecticide resistance and species diversity in *Anopheles gambiae* s.l. in Côte d’Ivoire” (Manuscript Number: PONE-D-24-56516).

We have carefully addressed all comments and suggestions from the reviewers and editorial team. All revisions have been incorporated in the main manuscript, and a detailed response letter is attached for your consideration. This work was supported by a grant awarded to the Institut Pierre Richet / Institut National de Santé Publique, Côte d’Ivoire, from the Bill & Melinda Gates Foundation (OPP1210335) through the Pan African Mosquito Control Association (PAMCA), which aimed to strengthen malaria vector surveillance in Côte d’Ivoire. The funders had no role in study design, data collection and analysis, decision to publish, or preparation of the manuscript.

We are committed to maintaining high standards of scientific accuracy and transparency, and we hope that the revised version meets the expectations of the journal.

Thank you again for your time and valuable feedback. We look forward to your response.

Best regards,

Rosine Z. Wolie

Corresponding Author (on behalf of all authors)

**Response to reviewers and journal requirements**

**Responses to journal editorial requirements**

**1.** Please ensure that your manuscript meets PLOS ONE's style requirements, including those for file naming. The PLOS ONE style templates can be found at

https://journals.plos.org/plosone/s/file?id=wjVg/PLOSOne_formatting_sample_main_body.pdf and

<https://journals.plos.org/plosone/s/file?id=ba62/PLOSOne_formatting_sample_title_authors_affiliations.pdf>

**Responses 1:** We corrected the formatting of all section titles, updated the corresponding author asterisk, edited the inline equation, and revised the table titles, column headers, and row labels to comply with PLOS ONE guidelines.

**2.** In your Methods section, please provide additional information regarding the permits you obtained for the work. Please ensure you have included the full name of the authority that approved the field site access and, if no permits were required, a brief statement explaining why

**Responses 2:** We clarified in the Methods section that national ethical approval, together with written informed consent from community members, served as the official authorization to conduct mosquito collections at all study sites (Lines 206-208.).

**3.** Please note that funding information should not appear in any section or other areas of your manuscript. We will only publish funding information present in the Funding Statement section of the online submission form. Please remove any funding-related text from the manuscript.

**Responses 3**: We have removed the funding information from the main manuscript, as requested. It is now included only in the Funding Statement section of the online submission form (Lines 346).

**4.** Please note that funding information should not appear in any section or other areas of your manuscript. We will only publish funding information present in the Funding Statement section of the online submission form. Please remove any funding-related text from the manuscript.

**Responses 4**: This point duplicates the previous request. As stated above, we have removed the funding information from the main manuscript.

**5.** Thank you for stating the following financial disclosure:

“This work was supported by a grant awarded to the Institut Pierre Richet / Institut National de Santé Publique, Côte d’Ivoire, from the Bill & Melinda Gates Foundation (OPP1210335) through the Pan African Mosquito Control Association (PAMCA). The grant aimed to strengthen malaria vector surveillance in Côte d’Ivoire, specifically addressing the genetic diversity and gene flow data gaps in insecticide-resistant Anopheles gambiae.”

Please state what role the funders took in the study.  If the funders had no role, please state: "The funders had no role in study design, data collection and analysis, decision to publish, or preparation of the manuscript."

If this statement is not correct you must amend it as needed.

Please include this amended Role of Funder statement in your cover letter; we will change the online submission form on your behalf.

**Responses 5**: The funders had no role in study design, data collection and analysis, decision to publish, or preparation of the manuscript. We will include this statement in the cover letter, as requested.

**6.** In the online submission form, you indicated that “The data supporting the conclusions of this manuscript are included within the text and are available from the corresponding author upon reasonable request.”

All PLOS journals now require all data underlying the findings described in their manuscript to be freely available to other researchers, either 1. In a public repository, 2. Within the manuscript itself, or 3. Uploaded as supplementary information.

This policy applies to all data except where public deposition would breach compliance with the protocol approved by your research ethics board. If your data cannot be made publicly available for ethical or legal reasons (e.g., public availability would compromise patient privacy), please explain your reasons on resubmission and your exemption request will be escalated for approval.

**Responses 6**: We have revised the Availability of data and materials section in the manuscript to align with PLOS ONE data sharing policy. It now states that the data are included in the text and that the underlying datasets will be deposited in a public repository and made fully accessible upon acceptance.

**7.** We note that Figure 1 in your submission contain map/satellite images which may be copyrighted. All PLOS content is published under the Creative Commons Attribution License (CC BY 4.0), which means that the manuscript, images, and Supporting Information files will be freely available online, and any third party is permitted to access, download, copy, distribute, and use these materials in any way, even commercially, with proper attribution. For these reasons, we cannot publish previously copyrighted maps or satellite images created using proprietary data, such as Google software (Google Maps, Street View, and Earth). For more information, see our copyright guidelines: http://journals.plos.org/plosone/s/licenses-and-copyright.

We require you to either (a) present written permission from the copyright holder to publish these figures specifically under the CC BY 4.0 license, or (b) remove the figures from your submission:

 a. You may seek permission from the original copyright holder of Figure 1 to publish the content specifically under the CC BY 4.0 license.

 We recommend that you contact the original copyright holder with the Content Permission Form (http://journals.plos.org/plosone/s/file?id=7c09/content-permission-form.pdf) and the following text:

“I request permission for the open-access journal PLOS ONE to publish XXX under the Creative Commons Attribution License (CCAL) CC BY 4.0 (http://creativecommons.org/licenses/by/4.0/). Please be aware that this license allows unrestricted use and distribution, even commercially, by third parties. Please reply and provide explicit written permission to publish XXX under a CC BY license and complete the attached form.”

 Please upload the completed Content Permission Form or other proof of granted permissions as an "Other" file with your submission.

 In the figure caption of the copyrighted figure, please include the following text: “Reprinted from [ref] under a CC BY license, with permission from [name of publisher], original copyright [original copyright year].”

 b. If you are unable to obtain permission from the original copyright holder to publish these figures under the CC BY 4.0 license or if the copyright holder’s requirements are incompatible with the CC BY 4.0 license, please either i) remove the figure or ii) supply a replacement figure that complies with the CC BY 4.0 license. Please check copyright information on all replacement figures and update the figure caption with source information. If applicable, please specify in the figure caption text when a figure is similar but not identical to the original image and is therefore for illustrative purposes only.

The following resources for replacing copyrighted map figures may be helpful:

USGS National Map Viewer (public domain): http://viewer.nationalmap.gov/viewer/

The Gateway to Astronaut Photography of Earth (public domain): http://eol.jsc.nasa.gov/sseop/clickmap/

Maps at the CIA (public domain): https://www.cia.gov/library/publications/the-world-factbook/index.html and https://www.cia.gov/library/publications/cia-maps-publications/index.html

NASA Earth Observatory (public domain): http://earthobservatory.nasa.gov/

Landsat: http://landsat.visibleearth.nasa.gov/

USGS EROS (Earth Resources Observatory and Science (EROS) Center) (public domain): http://eros.usgs.gov/#

Natural Earth (public domain): <http://www.naturalearthdata.com/>

**Responses 7**: Thank you for this important clarification. The map was generated using QGIS v2.14.19 with GPS coordinates of the mosquito collection points. The base layers were obtained from the Centre de Cartographie et de Télédétection (CCT) of the Bureau National d’Études Techniques et de Développement (BNETD) in Côte d’Ivoire. All vector layers, including vegetation types and administrative boundaries, were digitized by the authors using publicly available sources. No copyrighted or proprietary maps (e.g., Google Maps) were used. This information has been added to the revised Methods section and the revised figure has been included in the resubmission (Lines 128-131).

**8.** Please include captions for your Supporting Information files at the end of your manuscript, and update any in-text citations to match accordingly. Please see our Supporting Information guidelines for more information: <http://journals.plos.org/plosone/s/supporting-information>.

**Responses 8**: We have added a Supporting information section at the end of the manuscript, including full captions for each supplementary figure, table, and document, in accordance with PLOS ONE guidelines (Lines 545-558).

**9.** Please review your reference list to ensure that it is complete and correct. If you have cited papers that have been retracted, please include the rationale for doing so in the manuscript text, or remove these references and replace them with relevant current references. Any changes to the reference list should be mentioned in the rebuttal letter that accompanies your revised manuscript. If you need to cite a retracted article, indicate the article’s retracted status in the References list and also include a citation and full reference for the retraction notice.

**Responses 9**: We have reviewed the reference list and confirm that it is complete and up to date. No retracted articles are cited in the manuscript.

**Responses to Reviewers’ comments:**

**Comments to the Author**

1. Is the manuscript technically sound, and do the data support the conclusions?

The manuscript must describe a technically sound piece of scientific research with data that supports the conclusions. Experiments must have been conducted rigorously, with appropriate controls, replication, and sample sizes. The conclusions must be drawn appropriately based on the data presented.

Reviewer #1: Yes

Reviewer #2: Yes

2. Has the statistical analysis been performed appropriately and rigorously?

Reviewer #1: Yes

Reviewer #2: Yes

3. Have the authors made all data underlying the findings in their manuscript fully available?

The [PLOS Data policy](http://www.plosone.org/static/policies.action#sharing) requires authors to make all data underlying the findings described in their manuscript fully available without restriction, with rare exception (please refer to the Data Availability Statement in the manuscript PDF file). The data should be provided as part of the manuscript or its supporting information, or deposited to a public repository. For example, in addition to summary statistics, the data points behind means, medians and variance measures should be available. If there are restrictions on publicly sharing data—e.g. participant privacy or use of data from a third party—those must be specified.

Reviewer #1: Yes

Reviewer #2: Yes

4. Is the manuscript presented in an intelligible fashion and written in standard English?

PLOS ONE does not copyedit accepted manuscripts, so the language in submitted articles must be clear, correct, and unambiguous. Any typographical or grammatical errors should be corrected at revision, so please note any specific errors here.

Reviewer #1: Yes

Reviewer #2: Yes

**5. Review Comments to the Author**

Please use the space provided to explain your answers to the questions above. You may also include additional comments for the author, including concerns about dual publication, research ethics, or publication ethics. (Please upload your review as an attachment if it exceeds 20,000 characters)

**Reviewer #1:** The manuscript is well structured and written.

My only comments are that it might have been a more valuable study if further investigation into the specifics of metabolic resistance was investigated e.g. which P450s are associated with the resistance recorded rather than the more general finding associated with the use of PBO.
Also, data on the susceptibility to newer insecticides that are being introduced for vector control uses such as chlorfenapyr, clothianidin and broflanilide might have been an interesting addition.

**Response to reviewer 1:**
We appreciate the reviewer’s positive feedback and thoughtful suggestions. While our study focused on phenotypic resistance and target-site mutations, we acknowledge the importance of investigating specific metabolic resistance mechanisms, such as P450 gene expression. This limitation is now noted in the Discussion as a key area for future research.

Regarding newer insecticides like chlorfenapyr and clothianidin and broflanilide our study focused on insecticides for which resistance markers were well-characterized at the time, to examine how phenotypic resistance and known genetic markers are distributed across species and ecological zones. These markers are increasingly used to inform insecticide deployment and resistance management strategies. We also recognize the importance of evaluating susceptibility to these newer compounds, and this has been highlighted as a priority in our future research directions.

**Response to reviewer 2:**

**Reviewer #2**: This study is about mosquito diversity and the distribution of resistance mechanisms among malaria vectors in three ecological areas in Côte d’Ivoire.
The overall manuscript was very well written in easy English, with good results and important recommendations.
I only have minor reviews which if addressed could help the readers to better understand the study.

**Minor reviews:**

67 Please rephrase this sentence to make clearer when PBO was conducted

**Response 1**:

We thank the reviewer for this helpful observation. The sentence has been revised in the Abstract section and can be found at lines 66- 68 of the revised manuscript.

69-70 ‘target site mechanisms (Kdr L1014F and Ace-1R G119S) determined using PCR’ is there a ‘were’ that is missing?

**Response 2**:

We appreciate the reviewer’s attention to clarity. The sentence has been revised, and the updated version appears in the Abstract at line 71.

75 please delete ‘collected’

**Response 3**:

We thank the reviewer for this suggestion. The word “collected” has been removed, as recommended in the line 76.

76-77 ‘in the savannah area (97-100% of collected mosquitoes)’ and in all the result part of the background please change the range to the mean of the proportion with the confidence interval (CI). For example ‘in the savannah area (97.9%, 95% CI [96.2-99.7]) or simply rephrase

**Response 4**:

We have replaced the percentage ranges with mean values and 95% confidence intervals in the Abstract and can be found at line 77-83.

96-97 please add some references about the seven species

**Response 5**:

Thank you to the reviewer. A supporting reference has been added at line 102 and in the References section (lines 380-381).

99 Please write some key findings of these authors in the text

**Response 6**:

Thank you. Key findings from the cited studies have been added to contextualize species variation and distribution at lines 99-102.

108 the introduction is well written however it lacks some related literature that had already shown by their results the importance of addressing insecticides resistances patterns according to species level in Côte d’Ivoire and other country of Africa. Please add some of them. It is not clear what the authors mean by ‘adequately’, please be more precise

**Response 7**:

We thank the reviewer for this valuable suggestion. Relevant literature highlighting the importance of assessing insecticide resistance patterns at the species level in Côte d’Ivoire has now been added at lines 109-112. We have also clarified the intended meaning of “adequately” to better reflect the novelty of our study at lines 112-115.

109-110 add some references from the country of study

**Response 8**:

We thank the reviewer for this helpful suggestion. References from studies conducted in Côte d’Ivoire have now been added to strengthen this statement (line 117)

100, 122, 167 italic for malaria vector name Anopheles, please check throughout the paper

**Response 9**:

Thank you for the observation. All malaria vector names, including Anopheles species have been italicized as required, and formatting has been checked throughout the manuscript.

**Method/Result**
In the method section, it is important to mention how long the CDC light trap were installed (one week, one month, two days per week for two months, …?). This allows the reader to know the frequency of collection that allowed the authors to have the number of mosquitoes mentioned in the part of the results and help in terms of reproducibility of the study

129-132: ‘Adult mosquitoes were collected using CDC light traps, which were set for two to three nights per study site’. Does it mean that for each of the six sites chosen for this study just one CDC light trap was installed? Were they installed simultaneously in the sites or in different period? When talking about the three different locations (132) the authors only mentioned ‘near a bed and outside on the veranda’ is there any other location missing? Does it mean that there were three CDC light traps per house? This part of the method needs more details

**Response 10**:

Thank you for the comment. We have clarified the mosquito collection procedures in the Methods (lines 144-147), specifying the number of traps, household rotation, and trap placement and timing to improve clarity and reproducibility.

162: the authors stated that 504 mosquitoes were chosen randomly to conduct species ID. The selection was random between each ecological region/ each method of capture? How did the authors proceed to avoid any bias? Please be more explicit

**Response 11**:

Thank you. We have clarified the sampling strategy in the Methods section (lines 178-180). Mosquitoes were randomly selected per sentinel site and per collection method (larval collection raised for bioassays or adult collection). The selection was conducted across 6 of 12 NMCP-designated sentinel sites at that sampling period, ensuring representative coverage of each ecological zone in alignment with the national surveillance framework.

173 the authors mentioned odds ratios however throughout the manuscript there was no mention of OR. Please have a look

**Response 12**:

Thank you. The reference to odds ratios was a leftover from an earlier draft and has been removed at the line 189.

179 please add a link for the software

**Response 13**:

The software link has been added in the Methods section at the line 196.

213-214 repetition of ‘both’ please delete one of them

**Response 14**:

Thank you. The redundant word “both” has been removed to improve clarity in the line 232.


**Discussion**
241-243 It was not the first time that hybrids were identified in Aboisso please check this reference <https://doi.org/10.1371/journal.pone.0297604>

**Response 15**:

Thank you for this important correction. We have amended the sentence in the discussion section (lines 261-267) to reflect that hybrid specimens have previously been reported in Aboisso.

243-247 you can review this part of the discussion considering the number of hybrids they found in this study https://doi.org/10.1371/journal.pone.0297604 or any other study that you can find from Aboisso, considering the date of collection and also the method of capture

**Response 16**:

Thank you. The sentence has been revised to acknowledge prior detection of hybrids in Aboisso (Kouamé et al., 2024) and to better contextualize their rarity in natural settings (Lines 261-269).

259 Please add other references as you mentioned many other parts of Côte d’Ivoire

**Response 17**:

Thank you. Additional references covering other regions of Côte d’Ivoire have been added to support this section (Line 284).

262-265. Anything about the low number of mosquitoes collected in the savannah region compared to the others? Don’t the authors think that the number of localities sampled by ecological regions could have influenced difference in terms of number of mosquitoes collected and the composition of mosquito species? Please address this either in the main discussion or in the limits of the study
I would like also the authors to address the limitations of the study regarding the period of collection July-October, because maybe the results would have been different when talking about species distribution in other periods of the year

**Response 18**:
Thank you. We have acknowledged the limited sampling period and unequal site distribution as potential limitations and edited the paragraph in the Discussion (lines 296-299).

487 Table 1. please add the stage of mosquitoes (larval and adult) in the title to make it more explicit

**Response 19**:
Thank you. The title of Table 1 has been revised to lines 505-506.

489 In the legend of the table 1 please be clearer about what type of significance the letters and numbers shown. For example, for each species in each column is the same letter shows that there is not a significant difference between method of capture? What about the numbers?

**Response 20**: Thank you. We have clarified the meaning of the superscript letters and numbers in the Table 1 legend to indicate the type of comparisons and significance (Lines 516-518).

500 `Table’ instead of ‘tableau’

**Response 21**:

Thank you. The term “tableau” has been replaced with “Table” as requested (Line 518).

501 there is a ‘t’ missing in adult ‘catches’ in the table 2

**Response 22**:

Thank you for pointing this out. The missing “t” has been corrected in the table 2.

503 Again as for the table 1, in the legend ‘For each mutation in each species, values in the same column sharing the same superscript letters do not differ significantly’ it is not clear what are the variables that the authors were addressing. Is it a significant difference between method of capture? Between ecological areas? Please address each parameter (letter/number) and add this information to the legend
Figure 2. instead of proportion (%) you can write proportion% (number)

Title of Figure 3. repetition of ‘synergist’
Legend of Figure 3. Please address the abbreviations as they are written in the figure. Repetition of what the errors bars indicate

**Response 23**:

We have clarified the legend of Table 2; the revised version appears at lines 521-523.

We thank the reviewer for the suggestion. The Y-axis has been kept as “Proportion (%)”, and the

For Figure 2, corresponding number of mosquitoes (N) is already indicated in parentheses on each bar for clarity.

The repetition of the word "synergist" in the title of Figure 3 has been corrected.

We have revised the legend of Figure 3 to eliminate redundancy and clarify the abbreviations as presented in the figure. The updated legend is included in the revised manuscript.

6. PLOS authors have the option to publish the peer review history of their article ([what does this mean?](https://journals.plos.org/plosone/s/editorial-and-peer-review-process#loc-peer-review-history)). If published, this will include your full peer review and any attached files.


If you choose “no”, your identity will remain anonymous but your review may still be made public.


**Do you want your identity to be public for this peer review?** For information about this choice, including consent withdrawal, please see our [Privacy Policy](https://www.plos.org/privacy-policy).

Reviewer #1: No

Reviewer #2: No

[NOTE: If reviewer comments were submitted as an attachment file, they will be attached to this email and accessible via the submission site. Please log into your account, locate the manuscript record, and check for the action link "View Attachments". If this link does not appear, there are no attachment files.]

While revising your submission, please upload your figure files to the Preflight Analysis and Conversion Engine (PACE) digital diagnostic tool, <https://pacev2.apexcovantage.com/>. PACE helps ensure that figures meet PLOS requirements. To use PACE, you must first register as a user. Registration is free. Then, login and navigate to the UPLOAD tab, where you will find detailed instructions on how to use the tool. If you encounter any issues or have any questions when using PACE, please email PLOS at [figures@plos.org](mailto:figures@plos.org). Please note that Supporting Information files do not need this step.
